# An uncommon treatment of totally extruded and lost talus: a case report

**DOI:** 10.1186/1752-1947-8-322

**Published:** 2014-09-29

**Authors:** Dragica Maja Smrke, Primož Rožman, Borut Gubina, Igor Frangež, Barbara Rejec Smrke, Zoran Marij Arnež

**Affiliations:** 1 Department of Traumatology, University Medical Centre Ljubljana, Zaloška 7 Ljubljana, Slovenia; 2 Blood Transfusion Centre of Slovenia, University of Ljubljana, Šlajmerjeva 6 Ljubljana, Slovenia; 3 Department of Urology, University Medical Centre Ljubljana, Zaloška 7 Ljubljana, Slovenia; 4 Department of Neursurgery, University Medical Centre Ljubljana, Zaloška 7 Ljubljana, Slovenia; 5 UCO Chirurgia plastica e Ricostruttiva, Ospedale di Cattinara, Via di Fiume 447 Trieste, Italy

**Keywords:** Extruded talus, Reimplantation, Microsurgical free flap transfer, Arthrodesis

## Abstract

**Introduction:**

Total extrusion and loss of the talus is a rare injury with a wide choice of appropriate treatment, but rarely resulting in a fully functional recovery. We report on an uncommon case, both for the severity of the injury and for the uncommon treatment due to the patient’s rejection of secondary surgery.

**Case presentation:**

We treated a 16-year-old Caucasian man with the most extreme variant of a totally extruded and lost talus, accompanied with complex injury of the soft tissues of the ankle and foot. The treatment included urgent microvascular foot reimplantation, microvascular muscle free flap transfer, and temporary fixation. This kind of injury should typically be treated by tibiocalcaneal arthrodesis. However, this was not performed, as after the successful early stages of the treatment he strongly objected to another surgery due to his fully functional status and the successful therapeutic results of our early treatment.

**Conclusions:**

The injury described in this case study would ordinarily be treated by amputation, but due to the well-executed treatment in the early stages after the injury, the outcome was satisfying. Surprisingly and against our expectations, the late results of the treatment were successful even without arthrodesis. He is now 37 years old and has a functional foot 21 years after the injury.

## Introduction

Total extrusion and loss of the talus following a Gustilo IIIc [1] high-energy injury of the lower leg is extremely rare and often accompanied with other injuries [2-7]. The choice of appropriate treatment remains controversial and may include amputation, primary tibiocalcaneal arthrodesis [6,8], talar body prosthesis [9] or total ankle arthroplasty [7,10].

On the other hand, there are various appropriate choices for successful treatment when an extruded talus is not lost. Wound lavage and debridement before reduction of the extruded talus should be performed immediately after the injury [2,3,11-13]. Repositioning of the extruded talus is important for restitution of ankle biomechanics and represents a worthy alternative to tibiocalcaneal arthrodesis despite the fact that complication rates of avascular necrosis and infection remain high [2-4,7,12,14,15]. Primary reimplantation of a totally extruded talus is recommended even for contamination or articulare damage, while osteonecrosis of the talus does not necessarily lead to an unsatisfactory result [2,3,5-7,12]. A tibiocalcaneal arthrodesis after a totally extruded talus is recommended as a second stage or as a salvage procedure with remarkably satisfactory results [7,11]. Several authors also report the successful reimplantation of a talus after complete open extrusion after Gustilo IIIa and IIIb injury [2]. A successful reimplantation was also reported for a case where the talus was lost and retrieved several hours after the accident [6].

## Case presentation

**Figure 1:**
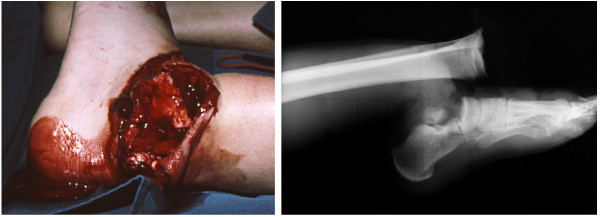
**a,b Gustilo IIIc high-energy injury of the ankle and foot with lost talus on admission.** Preoperative lateral radiograph of the left ankle and foot on admission (**a** - left) and skeletal disorders and lost talus (**b** - right).

**Figure 2:**
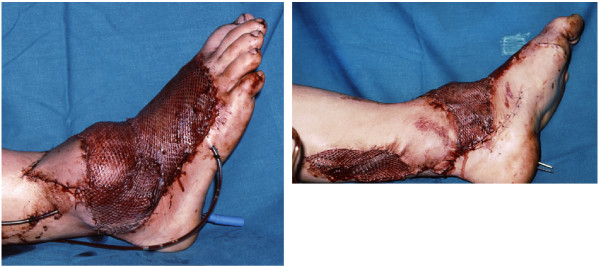
**End of the primary procedure with free flap transfer and temporary tibiocalcaneal arthrodesis with Kirschner wires.** Shown are the lateral view (left) and the medial view (right).

Twenty-one years ago a 16-year-old Caucasian man with open growth plates was injured in a motorbike accident. He was admitted directly to the emergency room in deep traumatic shock, with right sacroiliac joint luxation, diastasis of the symphysis of more than 10cm, and a complex injury of the left ankle and foot classified as Gustilo IIIc (Figure 1a). The injury consisted of a bimalleolar fracture with loss of the medial malleolus, extruded and lost talus, and fractures of the third to fifth metatarsal bones and the proximal phalanx of the great toe (Figure 1b). Small bone fragments seen in the wound were debrided. The left foot suffered incomplete amputation. The foot was pulseless and lacked sensitivity of the medial plantar skin.The operation started with external stabilization of the pelvis with a Hoffman external fixator followed by radical debridement of the ankle injury. The procedure resulted in a 15 × 10cm soft tissue defect on the dorsum of the foot and over the lateral malleolus exposing the extensor tendons and fractured metatarsals, extensor retinaculum, and a total defect of the talus. The anterior tibial vessels were badly contused over an area of more than 15cm and were considered not reconstructable. The posterior tibial vessels and the posterior tibial nerve were contused and cut at the level of the external malleolus. Provisional bone was fixed with two percutaneous Kirschner wires introduced through the calcaneus into the tibia. The posterior tibial vessels were repaired by suturing the artery and both concomitant veins in end-to-end fashion. The posterior tibial nerve was treated by an epineural suture. The foot was well perfused through the repaired posterior tibial vessels. The soft tissue defect was covered with a microvascular latissimus dorsi free flap transfer (Figure 2).

After two weeks the grafts were completely adherent and primary healing of all wounds was observed except on the great toe, which had to be amputated due to dry gangrene. There were no signs of infection, and he walked with crutches without bearing any weight. Kirschner wires were replaced with an AO external fixator two weeks after the accident.

Nine weeks after the injury the external fixator was replaced by a long leg plaster cast. He was given a Sarmiento walker plaster cast to be carried for the next two months. Later, he used a soft ankle orthosis.

**Figure 3:**
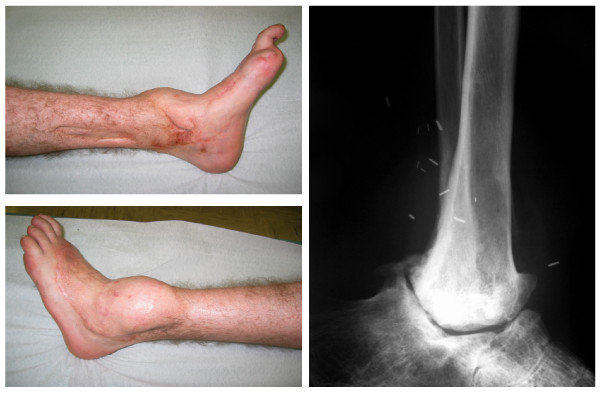
**Clinical status of the lower leg at the 19-year follow-up examination.** Medial and lateral views (left), and radiograph showing tibiocalcaneal pseudarthrosis (right).

Six months after the injury he was free from pain and completely able to bear weight. Radiographs showed a surprisingly good position of the distal tibio-fibular complex with respect to the superior calcaneal articulation. He spent the next half-year on physical therapy.After two years, he could walk and was fully able to bear weight using a cane in his right hand. A radiography showed there was no spontaneous arthrodesis. He refused the proposed tibiocalcaneal arthrodesis because he was satisfied with the results and did not want to undergo another surgical procedure. A radiograph of his ankle taken 19 years after the accident showed the convex tibial joint body and concave calcaneal joint body, with rather congruent articular surfaces (Figure 3).

**Figure 4:**
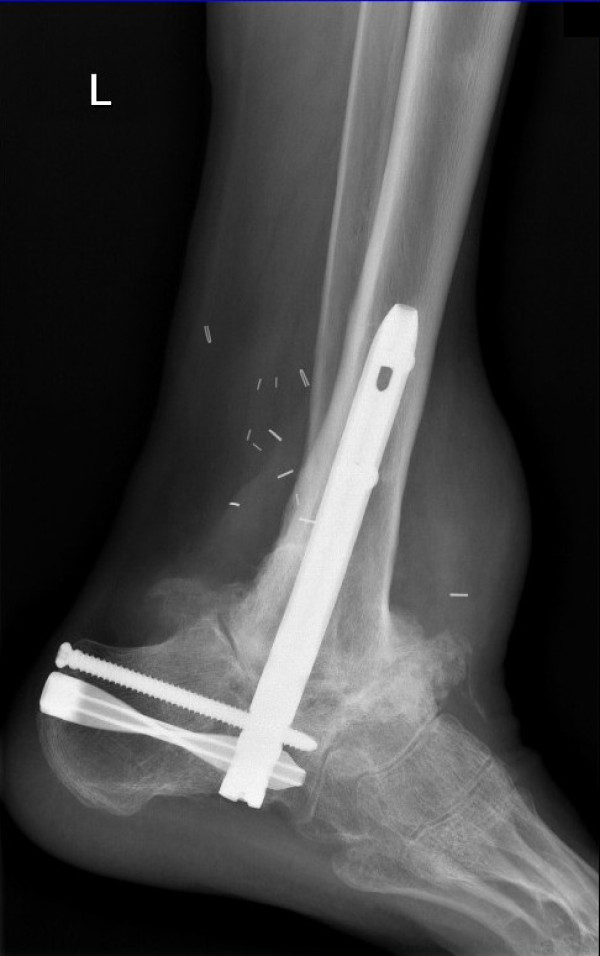
**Radiograph at 21-year follow-up.** The radiograph was taken six months after fixation with hindfoot arthrodesis nail and grafting.

Twenty years after the initial injury, he suffered another injury of the same leg with decompensation of the tibiocalcaneal nonunion. A stable fixation was performed with a hindfoot arthrodesis nail (Synthes) in combination with a composite graft consisting of autologous cancellous bone and allogeneic platelet gel [16] (Figure 4). A satisfactory reduction and stable fixation was achieved [17], leading to a complete recovery in six months.

At the time of writing of this case report, 22 years after the injury, he can walk with a slight limp without an aid and without pain. The shortening of the left leg remains at 2cm and is compensated by a shoe insert. He does not use any analgesics. He is capable of driving a car and works as an auto electrician, avoiding activities that involve lifting or carrying heavier objects.

## Discussion

We have presented a case with the most extreme variant of a totally extruded and lost talus accompanied with a semi-amputated foot. A review of the literature revealed only a few reports of similar cases, but none involving an adolescent in combination with foot reimplantation. In the first of these reports, the talus was lost and the foot was so severely damaged that the leg was amputated below the knee [18].

To avoid amputation, our primary treatment plan was first to perform a microsurgical reimplantation of the foot and use temporal fixation to avoid a pending infection that could be caused by a more complex treatment. This would be followed by a late tibiocalcaneal arthrodesis, which would provide viability of the microvascular flap and stabilize the general condition of our patient. After the initial treatment and successful healing of the soft tissues, his condition was stable. He was without pain and his injured leg could carry his full body weight. His tibiocalcaneal joint was fully functional. At this stage, during his hospitalization after his treatment, he was advised to complete the treatment by tibiocalcaneal arthrodesis. However, he has consistently refused any further treatment, referring to his fully functional condition and the absence of pain. We strongly suggested treatment by tibiocalcaneal arthrodesis after his hospitalization, during his rehabilitation and at several follow-up examinations, but we could not convince him, since he was satisfied with the outcome of the treatment and afraid of any potential complications from additional treatment.

Due to his explicit request, the tibiocalcaneal arthrodesis was not performed, resulting in an uncommon treatment that led to the self-formation of a new type of tibiocalcaneal joint and a surprisingly successful long-term result. His foot was functional for 20 years after the initial treatment, when minor injuries caused by a jump from a height of 0.7m led to decompensation of pseudarthrosis, which was treated with a standard arthrodesis procedure.

The procedure and long-term result cannot be directly compared with any from already published cases. There are, however, some similarities with cases where the talus was lost [8,10]. A case with a total ankle arthroplasty and a custom-built talar prosthesis resulted in a better outcome than in our case [10]. However, our patient suffered a more serious Gustilo IIIc injury. The primary use of a talar prosthesis for this type of injury is controversial because of possible high infection and complication rates [7]. In addition, implantation of a metal talar body prosthesis for an extruded and lost talus is a rarely performed approach [7,9,10]. Primary below-knee amputation as a salvage procedure was another option for our treatment, but we decided against it since our patient was a teenager at the time of his injury.

There are two possible drawbacks of our treatment. First, the recovery period would probably have been shorter if our patient had allowed the tibiocalcaneal arthrodesis soon after his initial clinical status had stabilized. We believe, however, that our decision to perform a primary reconstruction by the use of an urgent free flap transfer after radical debridement, blood vessel reconstruction, and temporary Kirschner wire fixation was appropriate because it avoided primary below-knee amputation and provided primary wound healing. Second, an early arthrodesis would also have prevented later injuries such as the decompensation of the tibiocalcaneal joint 20 years after the initial treatment.

## Conclusions

In comparison with the above-cited cases with open total talar dislocation, and considering the difficulty of the injury, the functional result of the reported case is exceptional. The optimal treatment for such cases has yet to be determined. Our case, which is uncommon because of our patient’s refusal of treatment by primary arthrodesis, surprisingly demonstrates that even without tibiocalcaneal arthrodesis the treatment can be successful and can grant functional results. This case report offers an alternative to present treatment options that should be considered when dealing with patients with severe foot injury and lost talus. The treatment carried out for our case is suggested for young patients with this type of injury in whom preservation of some movement may be preferable.

## Consent

Written informed consent was obtained from the patient for publication of this case report and any accompanying images. A copy of the written consent is available for review by the Editor-in-Chief of this journal.

## Competing interests

The authors declare that they have no competing interests.

## Authors’ contributions

DMS and ZA managed the case and wrote the manuscript. PR critically reviewed and edited drafts. BRS prepared the figures. BG and IF reviewed the manuscript and contributed the literature review. All authors read and approved the final manuscript.
